# Significance of CD133 expression in esophageal squamous cell carcinoma

**DOI:** 10.1186/1477-7819-11-51

**Published:** 2013-03-01

**Authors:** Hiroshi Okamoto, Fumiyoshi Fujishima, Yasuhiro Nakamura, Masashi Zuguchi, Yohei Ozawa, Yayoi Takahashi, Go Miyata, Takashi Kamei, Toru Nakano, Yusuke Taniyama, Jin Teshima, Mika Watanabe, Akira Sato, Noriaki Ohuchi, Hironobu Sasano

**Affiliations:** 1 Division of Advanced Surgical Science and Technology, Graduate School of Medicine, Tohoku University, 1-1 Seiryo-machi, Aoba-ku, Sendai 980-8574, Japan; 2 Department of Pathology, Tohoku University Hospital, 1-1 Seiryo-machi, Aoba-ku, Sendai 980-8574, Japan; 3 Department of Pathology, Graduate School of Medicine, Tohoku University, 2-1 Seiryo-machi, Aoba-ku, Sendai 980-8575, Japan

**Keywords:** AC133, Esophagus, Prominin-1, p16, p27, Stem cell marker

## Abstract

**Background:**

CD133 was recently reported to be a cancer stem cell marker and a prognostic marker for several tumors. However, few studies have investigated CD133 expression in esophageal squamous cell carcinoma (ESCC). Therefore, we examined whether CD133 could serve as a prognostic marker of ESCC and investigated the correlation between CD133 expression and the clinicopathological findings of ESCC patients and several markers.

**Methods:**

We studied 86 ESCC patients who underwent curative surgery without neoadjuvant treatment at Tohoku University Hospital (Sendai, Japan) between January 2000 and December 2005. We analyzed tissue specimens by immunohistochemical staining for CD133, p53, p16, p27, murine double minute 2 (MDM2), Ki-67, and epidermal growth factor receptor (EGFR).

**Results:**

Pathological tumor depth and tumor stage were significantly more advanced among CD133-negative patients than among CD133-positive patients. A log-rank test showed that CD133 immunoreactivity was significantly correlated with the overall survival of the patients (*P* = 0.049). However, multivariate analysis showed that it was not significantly correlated (*P* = 0.078). Moreover, CD133 was significantly positively correlated with p27 immunoreactivity (*P* = 0.0013) and tended to be positively correlated with p16 immunoreactivity (*P* = 0.057). In addition, p16 immunoreactivity was correlated with smoking history (*P* = 0.018), pathological lymph node status (*P* = 0.033), and lymphatic invasion (*P* = 0.018).

**Conclusions:**

This study indicated that CD133 immunoreactivity is a good predictor of prognosis in ESCC patients. In addition, CD133 may play a role in the regulation of tumor cell cycle through p27 and p16 in ESCC. At present, it thus remains controversial whether CD133 expression is a valid prognostic marker for ESCC. To elucidate this relationship, further investigations are required.

## Background

Prognosis or clinical outcome of esophageal squamous cell carcinoma (ESCC) has markedly improved over the last several decades, owing to advancements in medical treatment. However, in Japan, 11,867 people succumbed to this disease in 2010, and esophageal cancer was the seventh most common cause of cancer mortality in men (3.4% of the total cancer deaths in Japan) [1]. Various prognostic markers have recently been evaluated, including the stem cell marker CD133 (Prominin-1), which was reported to be a cancer stem cell marker for cancers of the brain [2], colon [3,4], prostate [5], liver [6,7], lung [8], kidney [9], ovaries [10], and skin [11,12]. It was also reported to be a marker of poor prognosis for cancers of the brain and spinal cord [13], colon [14], rectum [15], pancreas [16], breast [17], and stomach [18]. In addition, a potent cytotoxic drug, monomethyl auristatin F, which acts as an anti-CD133 antibody-drug conjugate for hepatocellular and gastric cancer cells, may be utilized to treat CD133-positive tumors [19]. To date, there have been limited studies of CD133 in esophageal cancer, and thus, the significance of CD133 in this form of cancer remains unclear. Therefore, we examined whether CD133 could serve as a prognostic marker of ESCC. In addition, we explored the correlation between CD133 expression and the clinicopathological findings of ESCC patients and the correlation between CD133 expression and the immunolocalization of several markers, such as p53, p16, p27, murine double minute 2 (MDM2), Ki-67, and epidermal growth factor receptor (EGFR), which are known as prognostic markers or tumor proliferation factors in ESCC [20-27].

## Methods

### Patients and tissue samples

A total of 86 consecutive ESCC patients, who underwent curative surgery without neoadjuvant treatment at Tohoku University Hospital (Sendai, Japan) between January 2000 and December 2005, were selected. All patients underwent thoracoscopic esophagectomy with two- or three-field node dissection, except for four patients who underwent pharyngo-laryngo-esophagectomy with one-field node dissection, six patients who underwent transhiatal esophagectomy with one-field node dissection, and eight patients who underwent esophagectomy by right thoracotomy.

The resected specimens and lymph nodes were fixed in 10% formalin, and representative sections were embedded in paraffin wax. The sections were histologically examined according to the Union for International Cancer Control (UICC) TNM (tumor, node, metastasis) classification (7th edition) system [28]. Patient survival time was determined from the date of surgery until death, recurrence, or the last follow-up examination. This study was approved by the ethical committee of the Tohoku University Hospital (Accession number 2011–596).

### Immunohistochemical staining and evaluation

Serial sections (4 μm thick), including the deepest area of the tumors, were deparaffinized in xylene, rehydrated in graded alcohol, and immersed in 3.0% hydrogen peroxide in methanol for 10 min at room temperature (RT) to inhibit endogenous peroxidase activity. For antigen retrieval, the slides for p53 were heated by microwave irradiation at 95°C for 15 min in 0.01 M citrate buffer (pH 6.0). The slides for p16, p27, MDM2, and Ki-67 were heated by autoclave at 121°C for 5 min in 0.01 M citrate buffer (pH 6.0). The slides for CD133 were autoclaved at 121°C for 5 min in Histofine antigen retrieval solution (pH 9.0, Nichirei Biosciences Inc., Tokyo, Japan). The slides for EGFR were incubated in 0.05% protease in Tris–HCl buffer (pH 7.6) at 37°C for 10 min. After washing three times for 5 min each in phosphate-buffered saline (PBS), the slides were incubated in 1% normal rabbit serum for 30 min at RT to reduce nonspecific antibody binding and were subsequently incubated at 4°C overnight with mouse monoclonal antibody against p53 (DO-7, Leica Microsystems, Bannockburn, IL, USA, diluted 1/100), p16 (G175-1239, BD Biosciences, Franklin Lakes. NJ, USA, diluted 1/100), p27 (SX53G8, Dako, Glostrup, Denmark, diluted 1/800), MDM2 (SMP14, Santa Cruz Biotechnology Inc., CA, USA, diluted 1/1000), Ki-67 (MIB-1, Dako, diluted 1/300), EGFR (31G7, Nichirei Biosciences Inc., dilution unknown, product code 413701), CD133 (AC133, Miltenyi Biotec, Auburn, CA, USA, diluted 1/10). The following day, the sections were washed three times for 5 min each in PBS, incubated with biotinylated anti-mouse immunoglobulin (Nichirei Biosciences Inc.), washed three times for 5 min each in PBS, and incubated with peroxidase-labeled streptavidin (Nichirei Biosciences Inc.) for 30 min at RT. The immunohistochemical signal was visualized with 3,3^′^-diaminobenzidine, and the slides were counterstained with Mayer’s hematoxylin, dehydrated in graded alcohol, and cleared in xylene. For CD133, omission of the primary antibody and substitution by nonspecific immunoglobulin (Mouse IgG1, Dako) at the same concentration were used as negative and isotype controls, respectively.

The sections were examined by two independent observers (HO and FF) who were blinded to patients’ clinical information. The proportion of positive nuclei in more than 1,000 tumor cells of more than three fields under a ×400 magnification microscope (Leica DM LB2) at the deepest area of each tumor was calculated for p53, p27, MDM2, and Ki-67. The proportion of nuclei and cytoplasm of tumor cells positive for p16 was evaluated. The proportion of membranes of tumor cells positive for EGFR was evaluated. The cut-off values for abnormal expression were as follows: p53, ≥10% [21]; p16, ≤5% [26]; p27, ≥10% [25]; MDM2, ≥20% [29]; and Ki-67, ≥30% [27]. An immunoreactive score (IRS) was used for the scoring of EGFR. The IRS is obtained by multiplying the intensity score (0, no staining; 1, faint staining; 2, moderate staining; 3, strong staining) by the extent score (0, none; 1, <10%; 2, 10 to 50%; 3, >50 to 80%; 4, >80%) and ranges from 1 to 12. An IRS of ≥6 was defined as positive for EGFR expression [20]. When evaluating CD133, the tumors were defined as positive and negative when >1% and ≤1% of the membranes and cytoplasm of all tumor cells were immunostained, respectively [13,30].

### Statistical analyses

Statistical analyses were performed using JMP Pro Version 9.0.2 (SAS Institute Inc., Cary, NC, USA). The correlation of factors was evaluated by the chi-square test, Fisher’s exact test, or Wilcoxon test, as appropriate. Survival curves were determined by the Kaplan-Meier method, and differences in survival between groups were compared by the log-rank test. The Cox proportional hazard model was used for multivariate analysis. A *P* value of <0.05 was considered statistically significant.

## Results

### Correlation between CD133 and clinicopathological findings of patients

**Figure 1:**
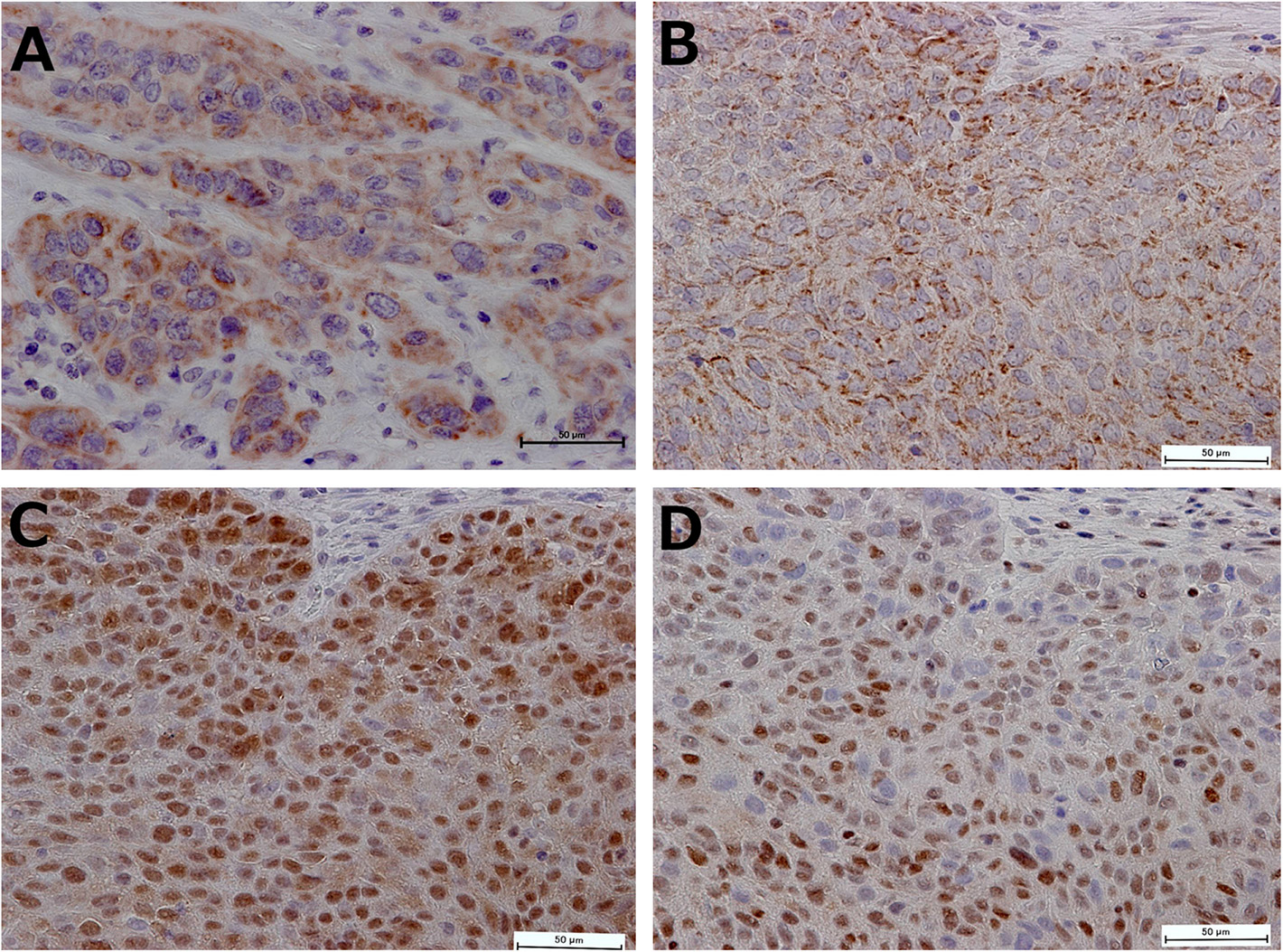
**Immunohistochemical staining of esophageal squamous cell carcinoma.** Tumor cells positive for CD133 **(A,B)**, p16 **(C)**, and p27 **(D)** expression (×400 magnification). In addition, **B**, **C**, and **D** were at the same site of the same tumor.

Table 1 summarizes the clinicopathological findings of the patients examined. The median follow-up time was 69.0 months (range, 1 to 149 months). The patients included 73 men and 13 women with a median age of 64 years (range, 37 to 81 years). The number of patients in each pathological stage was as follows: 20, pStageI; 28, pStageII; 33, pStageIII; and 5, pStageIV. There were five patients with M1 lymph nodes. Of the 86 patients, 38 (44.2%) were immunohistochemically positive for CD133 (Figure 1). pT and pStage were significantly more advanced among CD133-negative patients compared with CD133-positive patients (Table 1).

**Table 1:** Correlation between clinicopathological findings and CD133 status

		**Total (%)**	**CD133 expression**		***P***
			**Positive**	**Negative**	
			***n *= 38 (44.2%)**	***n *= 48 (55.8%)**	
**Age (years)**	Mean ± SD	63.9 ± 9.4	64.8 ± 10.9	63.2 ± 8.0	0.45
	(Range)	(37–81)	(37–9)	(44–81)	
**Sex**	Male	73 (84.9)	32 (84.2)	41 (85.4)	0.88
	Female	13 (15.1)	6 (15.8)	7 (14.9)	
**Smoking**	Absent	13 (15.1)	8 (21.1)	5 (10.4)	0.17
	Present	73 (84.9)	30 (79.0)	43 (89.6)	
**Location**	Cervix	5 (5.8)	3 (7.9)	2 (4.2)	0.59
	Upper	5 (5.8)	1 (2.6)	4 (8.3)	
	Middle	35 (40.7)	17 (44.7)	18 (37.5)	
	Lower	41 (47.7)	17 (44.7)	24 (50.0)	
**Histological type**	Well	17 (19.8)	9 (23.7)	8 (16.7)	0.68
	Moderate	56 (65.1)	23 (60.5)	33 (68.8)	
	Poor	13 (15.1)	6 (15.8)	7 (14.6)	
**pT**	pT1	28 (32.6)	19 (50.0)	9 (18.8)	0.017
	pT2	9 (10.5)	3 (7.9)	6 (12.5)	
	pT3	46 (53.5)	15 (39.5)	31 (64.6)	
	pT4	3 (3.5)	1 (2.6)	2 (4.2)	
**pN**	pN0	36 (41.9)	19 (50.0)	17 (35.4)	0.17
	pN1-3	50 (58.1)	19 (50.0)	31 (64.6)	
**pM**	pM0	81 (94.2)	35 (92.1)	46 (95.8)	0.65
	pM1 (LYM)	5 (5.8)	3 (7.9)	2 (4.2)	
**pStage**	pStageI	20 (23.3)	14 (36.8)	6 (12.5)	0.035
	pStageII	28 (32.6)	9 (23.7)	19 (39.6)	
	pStageIII	33 (38.4)	12 (31.6)	21 (43.8)	
	pStageIV	5 (5.8)	3 (7.9)	2 (4.2)	
**Lymphatic invasion**	Negative	34 (39.5)	13 (34.2)	21 (43.8)	0.37
	Positive	52 (60.5)	25 (65.8)	27 (56.3)	
**Venous invasion**	Negative	31 (36.1)	11 (29.0)	20 (41.7)	0.22
	Positive	55 (64.0)	27 (71.1)	28 (58.3)	

SD, standard deviation.

### Correlation between CD133 and other markers

Table 2 summarizes the correlation between expression of CD133 and expression of other molecular markers examined. CD133 and p27 expression were positively correlated (*P* = 0.0013), and CD133 and p16 expression tended to be positively correlated (*P* = 0.057) but did not reach statistical significance. No significant correlations were detected between expression of CD133 and expression of any other marker.

**Table 2:** Correlation between expression of CD133 and expression of other molecular markers

		**Total (%)**	**CD133 expression**		***P***
			**Positive**	**Negative**	
			***n *= 38 (44.2%)**	***n *= 48 (55.8%)**	
**p53**	Negative	29 (33.7)	13 (34.2)	16 (33.3)	0.93
	Positive	57 (66.3)	25 (65.8)	32 (66.7)	
**p16**	Negative	69 (80.2)	27 (71.1)	42 (87.5)	0.057
	Positive	17 (19.8)	11 (29.0)	6 (12.5)	
**p27**	Negative	37 (43.0)	9 (23.7)	28 (58.3)	0.0013
	Positive	49 (57.0)	29 (76.3)	20 (41.7)	
**MDM2**	Negative	66 (76.7)	31 (81.6)	35 (72.9)	0.35
	Positive	20 (23.3)	7 (18.4)	13 (27.1)	
**Ki-67**	<30	43 (50.0)	17 (44.7)	26 (54.2)	0.39
	≥30	43 (50.0)	21 (55.3)	22 (45.8)	
**EGFR**	Negative	46 (53.5)	23 (60.5)	23 (47.9)	0.24
	Positive	40 (46.5)	15 (39.5)	25 (52.1)	

### Correlations for other molecular markers

In terms of correlations between the other molecular markers and clinicopathological findings, p16 expression was correlated with smoking history (*P* = 0.018), pathological lymph node status (*P* = 0.033), and lymphatic invasion (*P* = 0.018) (Additional file 1). With regard to correlations among other molecular markers, p53 expression was positively correlated positively with Ki-67 expression (*P* = 0.0030) (Additional file 2).

### Survival analysis

**Figure 2:**
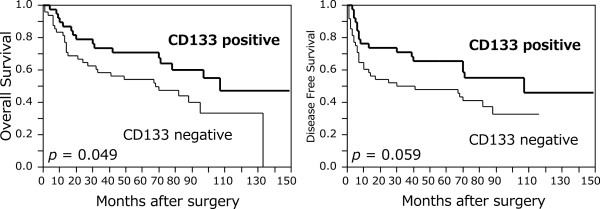
**Kaplan-Meier curves of patients with esophageal squamous cell carcinoma according to CD133 expression.** Overall survival was significantly longer in CD133-positive patients than in CD133-negative patients (*P* = 0.049). There was no significant correlation between disease-free survival and CD133 status (*P* = 0.059).

**Figure 3:**
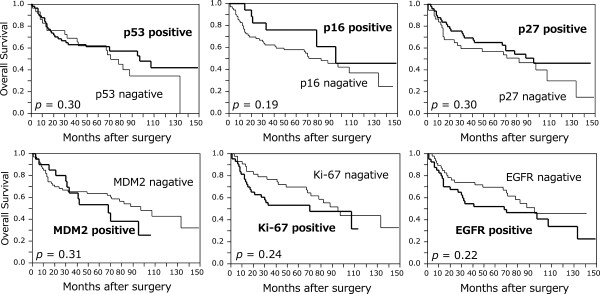
**Kaplan-Meier curves of patients with esophageal squamous cell carcinoma according to expression of the other markers.** No significant correlation between overall survival and the other markers was observed.

The 3- and 5-year survival rates of all patients examined were 65.0% and 61.5%, respectively. Results of univariate analysis of postoperative overall survival (OS) and disease-free survival (DFS) are summarized in Table 3. Overall survival was significantly correlated with pT, pN, pStage, and CD133 status, and was significantly longer in CD133-positive patients than in CD133-negative patients (*P* = 0.049) (Figure 2). No significant correlation between OS and the other markers was observed (Figure 3). Multivariate analysis demonstrated that pStage was a significant prognostic factor for OS and that pStage and tumor location were significant prognostic factors for DFS. Correlation between CD133 expression and patient survival did not reach statistical significance by multivariate analysis (Table 4).

**Table 3:** Univariate survival analysis of clinicopathological findings and expression of molecular markers

	**Variables**	**Number**	**Overall survival**		**Disease-free survival**	
			**5-year overall survival rate (%)**	***P***	**5-year disease-free survival rate (%)**	***P***
Age (years)	≤60	29	72.4	0.11	62.1	0.21
	>60	57	55.9		52.5	
Sex	Male	73	58.7	0.13	51.9	0.080
	Female	13	76.9		76.9	
Location	Cervix/upper	10	50.0	0.12	40.0	0.067
	Middle/lower	76	63.0		57.8	
Histological type	Well or moderate	73	61.5	0.64	57.4	0.36
	Poor	13	61.5		46.2	
pT	pT1/pT2	37	78.1	0.0012	70.0	0.0033
	pT3/pT4	49	49.0		44.9	
pN	pN0	36	80.6	0.0051	75.0	0.0078
	pN1-3	50	47.7		41.7	
pStage	pStageI/II	48	79.0	0.0002	72.8	0.0002
	pStageIII/VI	38	39.5		34.2	
Lymphatic invasion	Negative	34	70.2	0.086	58.4	0.29
	Positive	52	55.8		53.9	
Venous invasion	Negative	31	64.5	0.47	54.8	0.63
	Positive	55	59.8		56.2	
p53	Negative	29	62.1	0.30	55.2	0.55
	Positive	57	61.3		56.1	
p16	Negative	69	58.0	0.19	53.6	0.14
	Positive	17	76.0		63.7	
p27	Negative	37	56.8	0.30	54.0	0.49
	Positive	49	65.0		56.9	
MDM2	Negative	66	63.6	0.31	59.1	0.61
	Positive	20	53.3		59.2	
Ki-67	<30	43	69.8	0.24	62.8	0.27
	≥30	43	53.2		48.6	
EGFR	Negative	46	69.6	0.22	63.0	0.16
	Positive	40	52.0		47.1	
CD133	Negative	48	54.2	0.049	47.9	0.059
	Positive	38	70.8		65.5	

**Table 4:** Multivariate survival analysis of clinicopathological findings and expression of molecular markers

	**Variables**	**Hazard ratio**	**95% confidence interval**	***P***
**Overall survival**	pStageIII/IV	2.64	1.42-5.03	0.0020
	Lymphatic invasion positive	1.48	0.79-2.89	0.23
	CD133 negative	1.74	0.94-3.35	0.078
**Disease-free survival**	Male	2.09	0.84-6.99	0.12
	Cervix/upper	2.51	1.07-5.21	0.035
	pStageIII/IV	2.93	1.62-5.42	0.0004
	CD133 negative	1.73	0.96-3.24	0.071

## Discussion

CD133 was originally identified as a transmembrane glycoprotein in normal hematopoietic stem and progenitor cells [31] that participated in proliferation, self-renewal, and multilineage differentiation [32]. Furthermore, CD133 was recently used to identify putative cancer stem cells of several tumors [33]. According to several studies, CD133 was associated with tumor differentiation in several organs [16,34-36]. For example, Jiang *et al*. [36] reported that CD133 expression was increased in diffuse-type gastric cancers compared with intestinal-type cancers and was increased more so in poorly differentiated than in moderately or well differentiated gastric cancers. In addition, Feng *et al*. [35] reported that CD133 was negatively correlated with the cellular differentiation status of colon cancer cells. Finally, Fan *et al*. [34] reported that CD133 expression was correlated with well differentiated or moderately differentiated cholangiocarcinomas and that subcellular CD133 localization was correlated with the tumor differentiation status. In terms of ESCC, Hang *et al*. reported that CD133 expression was increased in well differentiated and moderately differentiated ESCCs compared with poorly differentiated ESCCs [30]. However, in our study, no correlation was detected between CD133 expression and tumor differentiation of carcinoma cells. We think that this was because there is a difference among pathologists or facilities regarding the histological evaluation of tumor differentiation status, and in addition, our study was small. On the other hand, CD133 expression correlated with p27 expression (*P* = 0.0013) and tended to correlate with the status of p16 immunoreactivity (*P* = 0.057). The relationship between CD133 and cell cycle regulators has remained unclear in esophageal cancer. There may be a correlation between CD133 and cell cycle pathways associated with the INK4 family or the CIP/KIP family of cyclin-dependent kinase inhibitors [37], but this possibility requires further investigation.

To the best of our knowledge, there are few reports that have investigated the effect of CD133 expression on survival of ESCC patients. Nakajima *et al*. [38] reported that CD133 expression in resected ESCC specimens following neoadjuvant chemoradiotherapy tended to be correlated with poor prognosis, but multivariate analysis did not produce a significant correlation. In contrast, CD133 expression was significantly correlated with poor response to neoadjuvant chemoradiotherapy. Hang *et al*. [30] reported that CD133 expression in resected ESCC specimens without preoperative treatment was not significantly correlated with prognosis. Our study revealed that OS was significantly longer in CD133-positive patients than in CD133-negative patients, as determined by log-rank test. One reason for this was that the tumors were significantly more advanced (according to their pStage classification) in CD133-negative patients than in CD133-positive patients. Although correlation between CD133 expression and patient survival did not reach statistical significance, as determined by multivariate analysis, CD133 immunoreactivity may have the potential to be a good predictor of prognosis in ESCC patients. With regard to other tumors, CD133 expression in non-small cell lung cancers [39,40], hepatocellular carcinomas [18], and pancreatic cancers [41] was not correlated with patient survival. Moreover, CD133-negative expression in cholangiocarcinomas was correlated with poor prognosis [34], which is similar to that revealed in our study. Further studies are needed to clarify this issue.

## Conclusions

In conclusion, this study demonstrated that CD133 immunoreactivity may have the potential to be a good predictor of prognosis in ESCC patients, and that CD133 may play a role in the regulation of tumor cell cycle through p27 and p16 in ESCC. At present, whether CD133 expression is a valid prognostic marker for ESCC remains controversial. To elucidate this relationship, further investigations are required, including verification of an evaluation method for CD133 immunoreactivity in ESCC.

## Consent

Written informed consent concerning the procedure of this study was obtained from all patients prior to study enrollment.

## Abbreviations

DFS: Disease-free survival; EGFR: Epidermal growth factor receptor; ESCC: Esophageal squamous cell carcinoma; IRS: Immunoreactive score; MDM2: Murine double minute 2; OS: Overall survival; PBS: Phosphate-buffered saline; RT: Room temperature; TNM: Tumor, node, metastasis; UICC: Union for International Cancer Control.

## Competing interests

The authors declare that they have no competing interests.

## Authors’ contributions

HO is the main author of this article. HO and FF conceived this study. FF and YN supervised the manuscript writing. MZ, YO, GM, TK, TN, YT, and JT contributed to the collection of clinical information and data analysis. HO and YT performed the experiments. HO and FF performed the pathological examination and immunohistochemical evaluation. MW, AS, NO, and HS reviewed the manuscript and revised it thoroughly. All authors have read and approved the final manuscript.

## Supplementary Material

Additional file 1 **Correlation between clinicopathological findings and p16 status.** p16 expression was correlated with smoking history (*P* = 0.018), advanced pN (*P* = 0.033), and lymphatic invasion (*P* = 0.018). Click here for file

Additional file 2 **Correlation between p53 status and Ki-67 status, MDM2 status.** p53 expression was positively correlated with Ki-67 expression (*P* = 0.0030). Click here for file
